# Examining non-syndromic autosomal recessive intellectual disability (NS-ARID) genes for an enriched association with intelligence differences^☆^

**DOI:** 10.1016/j.intell.2015.11.005

**Published:** 2016

**Authors:** W.D. Hill, G. Davies, D.C. Liewald, A. Payton, C.J. McNeil, L.J. Whalley, M. Horan, W. Ollier, J.M. Starr, N. Pendleton, N.K. Hansel, G.W. Montgomery, S.E. Medland, N.G. Martin, M.J. Wright, T.C. Bates, I.J. Deary

**Affiliations:** a Centre for Cognitive Ageing and Cognitive Epidemiology, University of Edinburgh, Edinburgh, UK; b Department of Psychology, University of Edinburgh, Edinburgh, UK; c Centre for Integrated Genomic Medical Research, University of Manchester, Manchester, UK; d Aberdeen Biomedical Imaging Centre, University of Aberdeen, UK; e Centre for Clinical and Cognitive Neurosciences, Institute Brain, Behaviour and Mental Health, University of Manchester, Manchester, UK; f QIMR, Berghofer Medical Research Institution, Brisbane, Australia

**Keywords:** Genetics, Intellectual disabilities, Gene set analysis, GWAS

## Abstract

Two themes are emerging regarding the molecular genetic aetiology of intelligence. The first is that intelligence is influenced by many variants and those that are tagged by common single nucleotide polymorphisms account for around 30% of the phenotypic variation. The second, in line with other polygenic traits such as height and schizophrenia, is that these variants are not randomly distributed across the genome but cluster in genes that work together. Less clear is whether the very low range of cognitive ability (intellectual disability) is simply one end of the normal distribution describing individual differences in cognitive ability across a population. Here, we examined 40 genes with a known association with non-syndromic autosomal recessive intellectual disability (NS-ARID) to determine if they are enriched for common variants associated with the normal range of intelligence differences. The current study used the 3511 individuals of the Cognitive Ageing Genetics in England and Scotland (CAGES) consortium. In addition, a text mining analysis was used to identify gene sets biologically related to the NS-ARID set. Gene-based tests indicated that genes implicated in NS-ARID were not significantly enriched for quantitative trait loci (QTL) associated with intelligence. These findings suggest that genes in which mutations can have a large and deleterious effect on intelligence are not associated with variation across the range of intelligence differences.

## Highlights

• Only three loci have been associated with intelligence.
• In traits such as height common variants are found in the same genes as rare variants.
• We hypothesise that intelligence may also follow this trend.
• We examine genes where rare variants can produce large deleterious effects on IQ.
• No enrichment was found for the non-syndromic intellectual disability gene set.

## 1 Introduction

The general factor of cognitive ability, termed general intelligence (or *g*), accounts for around 40% of the variation in any battery of cognitive tests which spans multiple cognitive domains (Carroll, 1993). It is also predictive of matters of importance such as educational and occupational outcomes, as well as disease status including cardiovascular disease and mortality (Deary, 2012). Population based studies using common single nucleotide polymorphisms (SNPs) as measured in Genome Wide Association Studies (GWAS) of intelligence indicate that common SNPs account for at least one third of phenotypic variation in intelligence differences (Benyamin, B., et al., 2013, Davies, G., et al., 2011, Kirkpatrick, R. M., et al., 2014, Marioni, R. E., et al., 2014a). These studies have, however, identified only three SNPs, at independent loci, at the genome-wide levels of significance required, and these jointly account for a very small amount of intelligence variation (Davies et al., 2015). Therefore, many genetic variants related to intelligence differences are yet to be found, suggesting that an alternative discovery strategies would be of value.

In traits such as height (Wood et al., 2014) and schizophrenia (Schizophrenia Working Group of the Psychiatric Genomics Consortium, 2014), where very large samples been assembled, the variants discovered have been shown to cluster in a much smaller number of biological systems. This in turn suggests that if such pathways could be identified for intelligence, they would present much richer candidates for gene discovery. More generally, this approach, of incorporating information from neurobiology or related phenotypes to prioritise specific regions of the genome, is termed pathway analysis or more accurately as gene-set analysis. Gene-set analysis can increase the power to detect an association by summing the variance captured by many SNPs (Hill, W. D., et al., 2014b, Liu, J. Z., et al., 2010, Torkamani, A., et al., 2008) and decreasing the number of tests made. Some progress has been made in this direction, for instance by examining the role of genetic variation in systems such as the *N*-methyl-D-aspartate receptor complex (NMDA-RC), linked to learning, it was shown that this system is also involved in intelligence differences (Hill et al., 2014a). This increase in statistical power makes gene-set analysis a powerful tool with which to seek replicable findings regarding the molecular genetic underpinnings of intelligence.

The rationale for the present study is that, like other complex traits such as height (Wood et al., 2014) and body mass index (Locke et al., 2015), mutations that result in large deleterious effects in a trait can occur in the same genes underlying the normal variation of a trait. To date many genes have been associated with intellectual disability (de Ligt, J., et al., 2012, Ellison, J. W., et al., 2013, Veltman, J. A. and Brunner, H. G., 2012) providing a number of candidate genes for analysis. In the case of intelligence, we exploit the existing knowledge of non-syndromic autosomal recessive intellectual disabilities (NS-ARID) (Musante & Ropers, 2014) to examine if these same genes are enriched for quantitative trait loci (QTL) associated with variation in the normal range of intelligence differences.

Intellectual disability (ID) is characterised by a significant impairment in cognitive ability. Within the DSM V (American Psychiatric Association, 2000), for a diagnosis of ID to be made, symptoms should be present before the age of 18 years, and IQ should be less than 70 (i.e., more than two standard-deviations below the population mean in the typical scoring system for IQ that use a mean of 100 and standard-deviation of 15). ID can be further divided into syndromic and non-syndromic forms. In syndromic ID, cognitive deficits are caused by identified medical problems such as phenylketonuria or foetal alcohol exposure. Non-syndromic ID is characterised by a lack of known pathology.

Genes associated with NS-ARID therefore form targets for understanding normal variation in intelligence, because they represent genes which, when mutations arise, can produce variation in cognitive ability without the presence of neurological abnormalities. Jointly, if normal variation in IQ is associated with genes which themselves can cause large changes in intellectual ability when mutated, then we should find excess association in these genes compared to genes outside this set.

To date, forty genes have been implicated in NS-ARID (Musante & Ropers, 2014). Eight of these (*PRSS12*, *CRBN*, *CC2D1A*, *GRIK2*, *TUSC3*, *TRAPPC9*, *ZC3H14*, *MED23*) were found by examining consanguineous families suffering with NS-ARID. The remaining 32 (*ADK*, *ADRA2B*, *ASCC3*, *ASCL1*, *C11orf46*, *TTI2*, *RABL6*, *CASP2*, *CCNA2*, *COQ5*, *EEF1B2*, *ELP2*, *ENTPD1*, *FASN*, *HIST3H3*, *INPP4A*, *MAN1B1*, *NDST1*, *PECR*, *PRMT10*, *PRRT2*, *RALGDS*, *RGS7*, *SCAPER*, *TRMT1*, *UBR7*, *ZCCHC8*, *ZNF526*, *CRADD*, *KIAA1033*, *ST3GAL3*, and *ZNF526*), were identified using next-generation whole exome sequencing. This group of 40 genes was used as the statistical unit of association to examine whether common genetic variation in genes associated with NS-ARID were associated with intelligence.

It is widely accepted that genes typically exert their effects as a part of pathways or networks of several genes each contributing to the activity of a biological system (Schadt, 2009). We therefore supplemented the core set of 40 NS ARID genes identified in (Musante & Ropers, 2014), by incorporating additional genes known to lie in pathways defined by these 40 core genes. In so doing, we hoped to increase our power to elucidate the mechanisms involved in intelligence (Lee et al., 2012b). The large (2 standard deviation of 30 IQ points) effect sizes associated with mutations in the NS-ARID gene set (Musante & Ropers, 2014) indicate how crucial a role these gene play in brain development and function. It is possible, therefore, that genes with such potentially catastrophic effects are under strong purifying selection to exclude all functional variation due to the crucial role these gene play. Indeed, evolutionary theories indicate that genetic variation resulting in a large reduction of a trait related to fitness, such as intelligence, will be subject to negative selection resulting in such variants remaining at a low frequency (Marioni et al., 2014b). This would mean that, although (rare) mutations in the NS-ARID gene-set can produce large deleterious effects on intelligence, common variation in the same genes might not be involved in variation in the normal range. If this is the case, we might expect to find no enrichment for normal variation in IQ in this set.

However, the mutations in the genes responsible for NS-ARID can also be viewed as causing variation in the function of the biological systems of which they are a part. Whereas mutations in the genes of the NS-ARID set lead to a large effect in the biological mechanisms they are in, common genetic variation throughout the rest of the system could result in more minor perturbations, which may underlie smaller decrements in cognitive ability.

To quantify the biological relationships between the 40 genes in the NS-ARID set, a statistical text-mining analysis tool was used in the present study, Gene Relationships Across Implicated Loci (GRAIL) (Raychaudhuri et al., 2009). This information was used to mine Gene Ontology (GO) (Ashburner et al., 2000) to extract gene sets indicated by the relationships between the genes of the NS-ARID set. By prioritising gene-sets linked to the shared function of the 40 NS-ARID gene-sets, statistical power can be kept high as only sets presumed relevant to intelligence would be tested.

There were four goals to this series of analyses. Firstly, the common SNPs in the genes of the NS-ARID set were analysed to determine if there was an association with the normal range of intelligence in a GWAS data set. Secondly, the effects of multiple SNPs were combined into a gene-based statistic to test the hypothesis that individual members of the NS-ARID gene set show an association with intelligence. The third aim was to determine if, in a GWA study of intelligence, the most significant SNPs are preferentially located in the NS-ARID gene set. The fourth aim was to prioritise gene sets based on functional relationships of the genes of the NS-ARID gene set and to test these additional functionally-related sets for an enriched association with intelligence.

## 2 Methods

### 2.1 Participants

Five independent cohorts forming the Cognitive Ageing Genetics in England and Scotland (CAGES) consortium were used. The individual cohorts used were the Lothian Birth Cohorts of 1921 and 1936 (LBC1921 and LBC1936)(Deary, Gow, Pattie, & Starr, 2012), the Aberdeen Birth Cohort of 1936 (ABC1936)(Whalley et al., 2011), and the Manchester and Newcastle Longitudinal Studies of Cognitive Ageing Cohorts (Rabbitt et al., 2004). This gave a combined sample size of 3511 healthy older individuals who live independently within the community.

Most of the LBC1921 took part in the Scottish Mental Survey 1932 (Deary, I. J., et al., 2009, Deary, I. J., et al., 2004, Scottish Council for Research in Education,, 1933). Individuals were identified and contacted through their general practitioner at around age 79 (M = 79.1, SD = 0.6 years). A total of 550 (316 female) of those individuals, living in Edinburgh and the surrounding Lothian regions, consented to recruitment to the LBC1921 cohort. Following informed consent, venous whole blood was collected for DNA extraction. Ethical approval was granted from the Lothian Research Ethics Committee.

Most of the LBC1936 (Deary et al., 2007) took part in the Scottish Mental Survey 147. This cohort was identified and contacted through their general practitioner at around age 70 (M = 69.5, SD = 0.8 years). A total of 1091 (543 female) of those individuals, living in Edinburgh and the surrounding Lothian regions, consented to recruitment to the LBC1936 cohort. The LBC1936 cohort is composed of healthy individuals who live independently within the community. Following informed consent, venous whole blood was collected for DNA extraction. Ethical approval was granted from Scotland's Multicentre Research Ethics Committee and the Lothian research Ethics Committee.

Most of the ABC1936 (Whalley et al., 2011) took part in the Scottish Mental Survey 1947. These individuals were identified and contacted through their general practitioner at around age 64 (M = 64.6, SD = 0.9 years). A total of 498 (255 female) of those individuals, now living in Aberdeen and the surrounding Grampian regions, consented to recruitment to the ABC1936 cohort. Following informed consent, venous whole blood was collected for DNA extraction. The Grampian Research Ethics Committee granted ethical approval.

The Manchester and Newcastle Longitudinal study of Cognitive Ageing Cohorts (Rabbitt et al., 2004) started in 1983/84; 6063 (4238 female) participants were examined over a 20-year time span. The ages range from 44 to 93 years (median 65). DNA was extracted following informed consent from 805 (572 females) participants from Manchester and 758 (536 female) of those from Newcastle. Ethical approval was granted from the University of Manchester.

A replication sample was formed using a total of 2062 participants (1093 female) of the Brisbane Adolescent Twin Study (BATS)(Wright & Martin, 2004) as well as those who have had cognitive phenotypes collected as a part of neuroimaging and cognitive studies (de Zubicaray, G. I., et al., 2008, Wright, M. J., et al., 2001). The sample was drawn from 928 families that included 339 monozygotic twin pairs and a single set of monozygotic triplets. Participants ranged in age from 15.4–29.6 years (mean = 16.6, SD = 1.5 years). DNA was extracted following informed consent. These studies were approved by the Human Research Ethics Committee at QIMR Berghofer and the institutional ethics boards of the University of Queensland and the Wesley Hospital.

### 2.2 Cognitive phenotypes

Fluid cognitive ability (*gf*), crystallised cognitive and general cognitive ability were all tested for association with the NS-ARID gene set. Fluid ability represents an individual's ability to process information on-the-spot; test items measuring *gf* typically make use of novel information, and tend not to be solve-able by using general knowledge (Horn, 1994). In each of the three Scottish cohorts *gf* was derived separately using the raw scores from each test. First, a principal components analysis was used and a score on the first unrotated component was extracted using regression. Second, for each component the standardised residuals were extracted from each model using age and sex as the predictor variables and the first unrotated component as the outcome variable.

In the LBC1921 cohort *gf* was derived from four tests. The Moray House Test (Scottish Council for Research in Education, 1933), Raven's Standard Progressive Matrices (Raven, Court, & Raven, 1977), phonemic Verbal Fluency (Lezak, Howieson, & Loring, 2004), and the Wechsler Logical Memory test (Wechsler, 1987) scores. In LBC1936 six tests taken from The Wechsler Adult Intelligence Scale III^UK^ (WAIS-III^UK^) were used. These were the Digit Symbol Coding, Block Design, Matrix Reasoning, Digit Span Backwards, Symbol Search, and the Letter–Number Sequencing tests (Wechsler, 1998). To derive *gf* in ABC1936, four tests were used: the Rey Auditory Verbal Learning Test (Lezak et al., 2004), the Uses of Common objects (Guildford, Christensen, Merrifield, & Wilson, 1978), Raven's Standard Progressive Matrices (Raven et al., 1977), and the Digit Symbol (Wechsler, 1981) from the WAIS revised (WAIS-R).

In order to derive *gf* in The Manchester and Newcastle Longitudinal study of Cognitive Ageing Cohorts, age and sex were first controlled for using residualisation. The standardised residuals from each test were then used in a maximum likelihood factor analysis where a general factor was extracted using regression and missing data points were imputed by sampling the posterior distribution of factor scores using Mplus (Muthen, Asparouhov, & Rebollo, 2006). The tests used for *gf* in the English cohorts were the two parts of the Alice Heim test 4 (Heim, 1970) and the four subtests of the Culture Fair Test (Cattell & Cattell, 1960).

Crystallised ability reflects an individual's level of acquired knowledge. Verbal tests such as vocabulary or reading ability provide a good measure of crystallised ability (Horn, 1994). In LBC1921, LBC1936 and ABC1936 the same test was used in each cohort, the National Adult Reading Test (NART)(Nelson & Willison, 1991). The Manchester and Newcastle Longitudinal study of Cognitive Ageing Cohorts used sections A and B from the Mill Hill Vocabulary Test (Raven, 1965). For each of the five cohorts, the raw scores from each test were used as the outcome variable in a linear regression, with age and sex used as predictors. The standardised residuals from these models formed the crystallised factor and were then carried forward to the genetic analyses.

The general factor of cognitive ability was derived in the same fashion as in Hill et al. (2014a). The same tests were used as for the construction of the fluid phenotype along with the addition of the total number of correct responses on the NART test. A principal components analysis was then carried out within each cohort and each participant's score on the first unrotated component was used to represent general cognitive ability. Next, the effects of age and sex were controlled for using regression. For the Manchester and Newcastle cohorts the effects of age and sex were regressed out of the standardised residuals for the gf and the crystallised ability factor. The mean of these two cognitive phenotypes was used to represent the general factor.

The BATS cohort used a performance IQ phenotype. This was derived using the Spatial and Objects Assembly tests scored from the Multidimensional Aptitude Battery (Jackson, 1984). The effects of age and sex were controlled for by regression.

### 2.3 Genotyping and quality control

The procedures implemented here have been described previously (Hill et al., 2014a). The 3782 participants in the CAGES consortium have been genotyped for 599,011 common SNPs using the Illumina610 QuadV1 chip (Illumina, San Diego, CA, USA). Following quality control, 549,692 SNPs remained from 3511 individuals. Individuals were excluded from the analysis following evidence of recent non-Caucasian descent, sex discrepancies, relatedness (at the level of second degree relatives), or a call rate of < 0.95. SNPs were included in the analyses if they had a call rate of > 0.98, a Hardy–Weinberg equilibrium test of P > 0.001 and a minor allele frequency of > 0.01. Population stratification was assessed using multidimensional scaling analysis (MDS) with outliers being removed. The first four MDS components were retained and included as covariates in subsequent GWA analysis. Imputation was performed within each cohort using MACH (v1.0.16) and the HapMap phase II CEU (NCBI build 36 release 22). SNPs were retained for analysis with an imputation quality score of greater than 0.3 and a minor allele frequency of > 0.005.

The genotyping and quality control procedures used on the BATS sample have been described previously (Medland et al., 2009). A total of 4391 individuals (twins and their non-twin siblings and parents) had DNA extracted from blood which was then genotyped using the Illumina Human 610-Quad chip (Illumina) and resulting in genotyping for 2062 twins and singletons (1,093 females) following quality control procedures. Individuals were excluded from the study if there was evidence of non-Caucasian descent or an unresolved gender discrepancy. SNPs which failed the call rate criteria of > 0.95, minor allele frequency > 0.01 and a Hardy–Weinberg equilibrium test of p > 0.000001 were excluded from the study (Medland et al., 2009). In order to control for the effects of population stratification, three multidimensional scaling components were included along with age and sex as covariates. Analyses were run on imputed dosage data (Release 6). The imputed data was generated from a set of SNPs common to 10 Illumina subsamples (19,275 individuals; 271,069 common SNPs). Imputation used HapMap release 22, build 36 as the reference panel. SNPs were dropped if the imputation quality score was < 0.3 and MAF < 0.01.

### 2.4 NS-ARID gene set

The 40 genes that were examined for an enriched association with cognitive abilities have each been previously linked with NS-ARID (Musante & Ropers, 2014) indicating that mutations in these genes have a large and deleterious effect on cognitive abilities with a loss of at least 30 IQ points. Whereas a sharp distinction between syndromic and non-syndromic ID is not always possible, the reduction in cognitive ability associated with the mutations in these genes is not merely the result of these mutations playing a causal role in other neurological disorders. Eight of these genes were identified using microsatellite based homozygosity mapping of large consanguineous families. These were followed up with mutation screening to identify the most likely gene responsible (Musante & Ropers, 2014). The remaining 32 were identified using Next-Generation Sequencing (NGS) methods including Whole Exome Sequencing (WES) and the enrichment and sequencing of exons from homozygous linkage intervals in consanguineous families (Musante & Ropers, 2014). Due to the overlap in rationale between this study and that of Franić et al. (2015) their set of 43 genes was analysed at the single marker, gene-based, and gene-set level. These results are found in the supplementary results sections and in Supplementary table 1.

### 2.5 Statistical analysis

Four levels of analyses were performed to interrogate the NS-ARID gene-set: Single marker analysis, gene level analysis, gene-set analysis, and systems level analysis.

### 2.6 Single marker analysis

Firstly, using data from the GWAS on fluid and crystallised ability, single marker analysis was conducted examining the 6,956 SNPs that were found within NS-ARID genes and within ± 50 kb of the known gene boundaries. Statistical significance was set to α = 7.188039e-06 (i.e., 0.05/6956).

### 2.7 Gene level analysis

Secondly, a gene-based statistic was derived by combining the effect of each SNP within a gene and the 50 kb boundary. Combining the effect of multiple SNPs has the potential to capture a greater proportion of variance which will lead to an increase in power (Hill, W. D., et al., 2014b, Liu, J. Z., et al., 2010). Gene-based statistics were derived using VEGAS (Liu et al., 2010) in which a test statistic is calculated from the sum of test statistics within a gene region with linkage disequilibrium (LD) being taken into account using the HapMap phase II CEU (NCBI build 36 release 22) reference panel for each gene and the 50 kb boundary. The statistical significance of this statistic is calculated using simulations. With 40 genes in the NS-ARID set the alpha level was 0.00125 (i.e., 0.05/40).

### 2.8 Gene-set analysis

Thirdly, a gene-set analysis was performed. In gene-set analysis, genetic data is aggregated from multiple genes that are united by sharing certain biological, functional, or statistical characteristics. This aggregation provides the advantage of reducing the multiple testing burden, as the whole gene set forms the statistical unit of association making it possible to detect small but consistent deviations from the chance level of association. Gene-set analysis has also been shown to be able to increase statistical power because, as is found in gene level analysis by contrast with single marker analysis, the effect of multiple SNPs is summed (Hill et al., 2014b). Gene-set analysis can be subdivided into self-contained testing and competitive testing. The difference between these two depends on the null hypothesis being tested. Self-contained tests examine if the a priori gene-set shows an association with the trait of interest, whereas competitive tests are used to show that the a priori gene-set shows a greater level of association compared to other gene sets. As there are more low p-values in a GWAS than would be expected under the null hypothesis, self-contained tests will inflate the type 1 error rate; for this reason competitive testing is recommended.

In order examine the NS-ARID gene set, INRICH (Lee, O'Dushlaine, Thomas, & Purcell, 2012a) was used. A gene-set analysis using INRICH proceeds through a number of steps. Firstly, regions of the genome showing evidence of association are identified using the clump function in PLINK (Purcell et al., 2007). These regions were derived by selecting SNPs, termed index SNPs, where the p-value is below 0.0005. Regions around these index SNPs are included by adding SNPs which are nominally significant, within 250 kb and correlated (in LD of r^2^ > 0.5 using the HapMap2 CEU reference panel) with the index SNP. This creates regions of the genome (genomic intervals) that show evidence of association that is independent of associations found in the other regions. Genomic intervals were excluded from subsequent analysis if they did not overlap within 20 kb (5′ or 3′) of any known gene according to the UCSC human genome browser hg 18 assembly. Secondly, a test statistic describing the level of association between the a priori gene-set and the phenotype is derived. This is defined by counting the number of times the independent genomic intervals overlapped with the a priori gene-set. The total number of times the independent intervals overlap with the gene-set is the test statistic. Thirdly, the statistical significance of this test statistic is determined using a competitive test. This is carried out by creating genomic intervals that contain the same number of genes, SNP density and LD structure as the independent genomic intervals derived using evidence of association with the phenotype. 10,000 permutations were used to derive an empirical p-value for the gene set defined as the proportion of permuted statistics that are equal to or exceed the observed gene set statistic.

### 2.9 Systems level analysis

The fourth analysis carried out aimed to quantify the biological relationship between the genes of the NS-ARID set and to use this knowledge to test the systems and pathways that reflect these processes for an association with intelligence. Here, Gene Relationships Across implicated Loci (GRAIL) (Raychaudhuri et al., 2009) was used to examine the 40 genes of the NS-ARID gene set and identify common cellular process or pathways. This was carried out using a text mining algorithm to derive a set of statistically significant keywords describing the relationship between the 40 NS-ARID genes. Using the a priori gene-set GRAIL can be used to identify a subset of genes that are more related than chance as well as to assign statistically significant keywords suggesting a pathway or system that unites the members of the gene-set. Importantly, this metric is derived without the use of the phenotype, meaning that potentially biased ideas about which pathways or biological functions influence the phenotype cannot dominate the analysis. Additionally, undocumented or distant relationships between the members of the gene-set can be indicated.

These keywords were derived using a database of 259,638 abstracts taken from PubMed before December 2006. This date was selected as it is prior to the mainstream application of GWAS, as abstracts detailing the regions identified by GWAS would be expected to confound the analysis by describing the NS-ARID gene set as being associated with NS-ARID. The GRAIL parameters applied were as follows: release 22/HG 18; HapMap population: CEU; Functional Data source PubMed Text (December 2006); Gene size Correction on; Gene lists; All human genes within the database.

Each of these abstracts was converted into a vector of word counts and, for each gene, a vector consisting of averaged word counts is derived. The relationship between any pair of genes is defined as the correlation between the two vectors of averaged word counts. This means that if two genes are described using the same words they will receive a high similarity score; however, they do not need to be mentioned in the same abstract in order to be classed as similar. After the relationship between the members of the gene-set has been examined, keywords are derived. These keywords are defined as those that have the greatest weight across all of the text vectors for the genes of the gene-set. Keywords are restricted to those that appear in > 500 abstracts and contain > 3 letters and no numbers.

Following the generation of the keywords, Gene Ontology (GO) (Ashburner et al., 2000) was mined. Here, the keywords derived by GRAIL to suggest pathways or systems common to the NS-ARID gene-set were used as search terms in GO. All gene-sets with at least five human genes were extracted and examined using INRICH to discover whether these showed significant overlap with the intervals generated from the GWAS data. As multiple gene-sets are being tested in this section of the study, the p-value generated for each gene-set will need to be corrected for the number of tests made. As the gene-sets are not independent, corrections such as the Bonferroni or false discovery rate will yield an overly conservative estimate of significance (Holmans et al., 2009), and so a bootstrap approach was used. Firstly, one of the 10,000 permuted interval sets was selected at random to serve as the observed interval set. Secondly, the statistical significance for the interval set serving as the observed data was derived as before by generating intervals across the genome and comparing the overlap with the gene-sets. Finally, the corrected p-value is the proportion of bootstrapped samples where the minimum gene p-value over all the gene-sets is at least as significant as the p-value for the gene-set being corrected for (Lee et al., 2012a). By using GRAIL to examine the functional relationship between the genes of the NS-ARID gene-set followed by GO to construct gene sets based on these shared functions, this series of analyses tests the hypothesis that the genes responsible for NS-ARID are functionally related to systems where common SNP variation can explain variation in intelligence.

## 3 Results

### 3.1 Single marker analysis

**Fig. 1:**
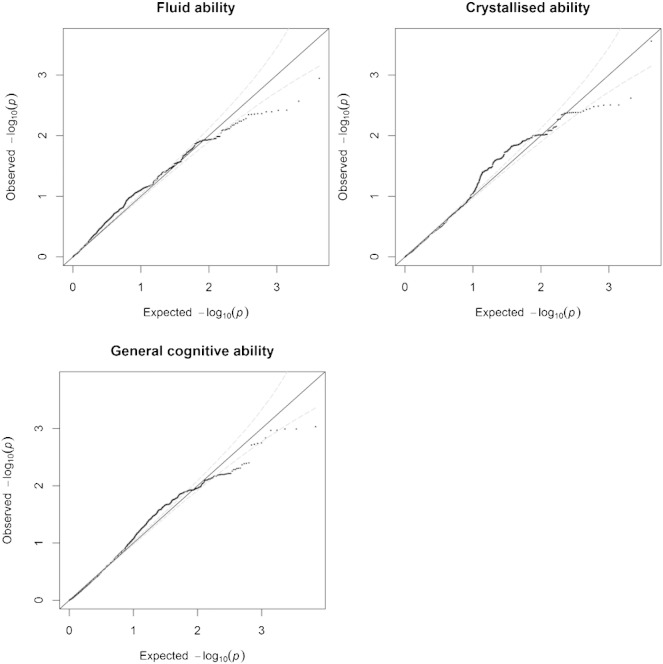
These qq plots show the full complement of 6,956 SNPs in fluid ability (top left), crystallised ability (right), and general cognitive ability (bottom left). The x-axis shows the expected distribution of − log10 p-values should they follow the null hypothesis of no association whereas the y-axis shows the observed values. Each point represents a single SNP. Points that deviate from the diagonal line indicate SNPs that deviate from the null hypothesis of no association. These plots indicate that, for both phenotypes, there is no deviation from that which would be expected under the null hypothesis of no association.

None of the 6,956 SNPs tested in either the fluid ability, crystallised ability, or general cognitive ability attained statistical significance where α = 7.188039e-06 (Fig. 1). Supplementary Tables 2, 3, and 4, show the most significant 50 SNPs for fluid, crystallised, and general cognitive ability.

### 3.2 Gene-based analysis

VEGAS (Liu et al., 2010) was used to examine the contribution each gene in the NS-ARID gene-set made to both fluid, crystallised, and general cognitive ability. No single gene-based statistic was significant at the adjusted alpha level of 0.00125 (see Table 1). Three nominally significant genes were found for *gf* (*CCNA2*, *C8orf41*, *ELP2*), one for crystallised ability (*ST3GAL3*), and four for general cognitive ability (*ST3GAL3*, *CCNA2*, *C8orf41*, *SCAPER*); these results are consistent with what would be expected under the null hypothesis.

### 3.3 Gene-set analysis

In order to conduct a gene-set analysis of the NS-ARID set using INRICH (Lee et al., 2012a), a series of LD independent genomic intervals were created. Using the clump function for the fluid phenotype, 407 genomic intervals were created, of which 248 overlapped within 20 kb of a known gene. Overlapping intervals were then merged leaving 176 LD independent intervals to be analysed for enrichment. For the crystallised ability, 403 intervals were produced with 221 overlapping known genes and the 20 kb boundary. Once overlapping intervals had been merged for the crystallised ability phenotype, 166 non-overlapping intervals were created and tested for an enriched association with the NS-ARID gene set. For general cognitive ability, the clump procedure left 398 genomic intervals, of which 222 overlapped with known genes. When overlapping interval had been merged there were 172 independent intervals taken forward for analysis. 10,000 permutations were used to assess statistical significance.

For *gf*, one member of the NS-ARID gene set, *TTI2*, overlapped with one of the LD independent genomic intervals on chromosome 8 spreading from 33398369 to 33503864. This overlap was not significant, p = 0.520. For crystallised ability and general cognitive ability none of the most significant regions in the GWAS overlapped with the NS-ARID gene set.

### 3.4 Systems level analysis

In order to assemble a list of gene sets that were most likely to be involved in variation in intelligence GRAIL was used to derive 18 keywords describing the relationship between the 40 NS-ARID genes (see Table 2). These keywords were then used as search terms to mine Gene Ontology, producing 180 gene sets which were then tested for an enriched association with *gf* and crystallised ability. Table 3 shows the most significant gene sets for fluid ability. The overlap between the most significant LD regions in the GWAS and GO:0006814, sodium ion transporters, was statistically significant after controlling for multiple tests. Table 4 shows the results for crystallised ability and Table 5 shows the most significant gene sets for general cognitive ability. Whilst the overlap with the 180 gene sets produced using GRAIL and Gene Ontology did not survive multiple correction, GO:0006354 is nominally significant in both *gf* and crystallised ability and this is partly due to the same genes, *POLR2B* on chromosome 5 and *POLR2E* on chromosome 19 being tagged by the LD independent intervals for both the fluid and the crystallised phenotypes.

### 3.5 Replication

In order to try and replicate the over-representation of the genes from the Sodium ion transport gene-set with fluid ability, the performance IQ phenotype from the BATS cohort was used. The same data processing pipeline was used to assemble independent intervals. Observed intervals from the BATS cohort overlapped with one gene from the Sodium ion transport gene-set, *SLC6A5.* This overlap was not statistically significant *p* = 0.922. None of the other nominally significant gene-sets contained genes which overlapped with the intervals of the BATS cohort and so no p-value could be derived.

## 4 Discussion

GWAS on intelligence have so far found three independent loci harbouring potentially causal variants (Davies et al., 2015). In the current study we sought to increase power to detect such variants by limiting the search to a smaller region of the genome. GWAS for quantitative traits, such as height, have shown that genetic variants contributing toward variance in the normal range are concentrated in the same genomic regions as rare variants of large effect (Wood et al., 2014). Using a set of forty genes where mutations are associated with large effects on intelligence, we examined if these same genes were also enriched for common variants of small effect, as would be predicted if the genetic architecture for intelligence followed the trend for other quantitative traits, such as height. No evidence for enrichment was found indicating that different regions of the genome are involved in the normal arrange of intelligence compared to the extreme ends of the distribution.

Four analysis strategies were used to examine whether genes involved in large deficits of cognitive ability also harbour common variants responsible for some of the variation in the normal range of intelligence differences. The first test examined each SNP using single marker analysis and found no evidence for a role in intelligence for any single SNP examined. The second strategy adopted was to sum the effect of multiple SNPs into a gene-based statistic using VEGAS (Liu et al., 2010) in an effort to capture a greater proportion of signal and so increase statistical power. Here, none of the 40 genes tested withstood correction for multiple comparisons. The third test examined the whole NS-ARID gene-set as the statistical unit of association. INRICH was used for this test and found that the most significant hits in the GWAS did not overlap with the genes found in the NS-ARID set more than would be expected by chance. This does not provide evidence that common variants in the genes involved in major deleterious deviations in cognitive ability also account for intelligence differences.

The lack of association between the NS-ARID SNPs, genes and gene-set raises the possibility that intellectual disability is genetically distinct from the normal variation of intelligence differences. Evidence to support this comes from a study conducted examining the siblings of children affected by severe mental retardation, classified as those whose IQ was < 50, and those with mild mental retardation, IQ 50-69 (Nichols, 1984). It was found that the siblings of those affected by severe mental retardation had an average IQ when compared to the population (mean = 103.4, SD = 12.1) and none of the siblings were suffering with any form of mental retardation. This contrasts with the siblings of children with mild mental retardation whose mean level fell below that of the population (mean = 84.8, SD = 18.1). In addition, 20.7% of these siblings also suffered with mental retardation.

The fourth analysis was conducted using GRAIL (Raychaudhuri et al., 2009) to quantify the relationship between the genes of the NS-ARID set with the goal of using this knowledge to examine the systems and pathways that reflect these processes to find a gene-set associated with intelligence. Whereas the genes of the NS-ARID gene-set (Musante & Ropers, 2014) may not be directly involved in the normal range of intelligence differences, they may be genes of particular importance and should variation occur here it may have deleterious consequences for any system that they are a part of. The goal of the GRAIL analysis was to identify the systems and processes they are a part of, as these may be more tolerant of functional variation and so may be involved in intelligence differences. The results of GRAIL identified 180 systems and processes which were then used to construct gene sets from Gene Ontology (Ashburner et al., 2000) following which they were examined for overrepresentation. One gene set, sodium ion transport, GO:0006814 remained significant for fluid ability after correction for the 180 gene-sets examined. This gene set is involved in the directed movement of sodium ions across the boundary of a cell by means of a transporter or a pore (Ashburner et al., 2000). Such actions are found in the nervous system in the form of the sodium–potassium pumps of the neuron. These pumps are responsible in establishing the resting potential of neurons where they serve to keep the concentration of sodium within the neuron low by moving against the gradient of electrostatic pressure. By the same means they also re-establish this gradient following depolarisation. This indicates a role for genetic variation of the neuron being involved in fluid cognitive abilities. However, this association failed to replicate in the younger BATS cohort, indicating that the initial significant result might be a type 1 error. It is also possible that the effect size in the discovery sample was overestimated (The winner's curse) (Ioannidis, 2008) meaning that the replication sample lacked the statistical power needed for replication.

The difference between the age of the discovery and replication cohorts may also have contributed toward the lack of replication. The genetic correlation between intelligence in old age and in childhood is estimated at 0.71 (SE = 0.101, *p* = 2.256e-12)(Hill, et al., in press), indicating that there is substantial pleiotropy between intelligence in childhood and old age. However, different genetic factors underlie cognitive ability in old age as indicated by the finding that the rs10119 variant in the *APOE*/*TOMM40* region has a greater deleterious effect on cognitive function as age increases (Davies et al., 2015).

Another finding was that the DNA-templated transcription, elongation gene-set (GO:0006354) was nominally significant in both the fluid and the crystallised phenotypes which correlate phenotypically at 0.58 in this sample. Whilst gene set analysis does not require the same genes to show an effect across two phenotypes for significance of the set to be established, in this instance two genes, *POLR2B* on chromosome 5 and *POLR2E* on chromosome 19, were found to tag SNPs with low p-values indicated by their presence in the LD independent intervals. The DNA-templated transcription, elongation gene set is described by Gene Ontology as being involved in the extension of the RNA molecule following the initiation of transcription and promoter clearance at DNA dependent RNA polymerase promoters through the inclusion of ribonucleotides catalysed by an RNA polymerase. Whilst this may indicate that the mechanisms involved in transcription, particularly elongation, are involved in cognitive abilities, it should be noted that, in the BATS sample, none of the genes from the DNA-templated transcription, elongation gene-set overlapped with any of the LD independent intervals.

The strengths of this study include the construction of a candidate gene set based on the rationale that, like other complex traits including height (Wood et al., 2014) and body mass index (Locke et al., 2015), common variants underlying normal variation in intelligence cluster in genes and pathways associated with rare variants of large effect. By including single marker analysis, gene-based analysis, gene set analysis and systems level analysis, a thorough interrogation of the NS-ARID set was performed. In addition, where statistical significance was found replication was sought.

The limitations of this study include the modest sample sizes used, as well as the use of Gene Ontology. Whilst mining Gene Ontology for gene sets using GRAIL represents a method to focus our investigation on potentially relevant gene sets, it may also omit causal pathways as gene-sets not implicated will be absent from the analysis. In addition, phenotypic heterogeneity may be a limitation, as different cognitive tests were used to derive a general factor of cognitive ability in each sample. However, it should be noted that the correlation between general factors derived from different sets of tests in the same sample is high (Davies, G., et al., 2015, Johnson, W., et al., 2004, Johnson, W., et al., 2008).

In conclusion, this study found no evidence that the genetic architecture of NS-ARID involves the same genes as those responsible for the normal range of intelligence differences. This indicates that NS-ARID is not the lower tail of the intelligence distribution, but is genetically distinct. In addition, there was tentative evidence that the sodium ion transporters may underlie genetic variation in fluid ability in older adults.

The following are the supplementary data related to this article.

Supplementary material Results using the gene set first analysed by Franić, et al. (2015). S1 shows the results of SNP based analysis, S2 and Table S1 show the results of gene-based analysis. S3 shows the results of the Franić, et al. (2015) gene-set when considering all the genes as the unit of association.

Table S2 The estimated effect size, standard error and P-values are shown for the most significant 50 SNPs for fluid ability. SNPs were mapped to genes based on their position in the UCSC hg18 annotation. The order that the cohorts appear in the direction of effect column is ABC, LBC1921, LBC1936, Manchester then Newcastle. A direction of effect of “?” indicates that the SNP failed quality control in that cohort.

Table S3 The estimated effect size, standard error and P-values are shown for the most significant 50 SNPs for crystallised ability. SNPs were mapped to genes based on their position in the UCSC hg18 annotation. The order that the cohorts appear in the direction of effect column is ABC, LBC1921, LBC1936, Manchester then Newcastle.

Table S4 The estimated effect size, standard error and P-values are shown for the most significant 50 SNPs for General ability. SNPs were mapped to genes based on their position in the UCSC hg18 annotation. The order that the cohorts appear in the direction of effect column is ABC, LBC1921, LBC1936, Manchester then Newcastle. A direction of effect of “?” indicates that the SNP failed quality control in that cohort.

## Figures and Tables

**Table 1:** Gene based analysis results for the 40 NS-ARID genes in the CAGES consortium. p-values reported are uncorrected.

Chr	Gene ID	nSNPs	Start	Stop	Fluid p-value	Crystallised p-value	General cognitive ability
1	*HIST3H3*	48	226679168	226679649	0.203	0.734	0.305
1	*RGS7*	804	239005439	239587101	0.983	0.997	0.998
1	*ST3GAL3*	233	43945804	44169418	0.378	**0.025**	**0.084**
2	*ADRA2B*	29	96142349	96145615	0.091	0.599	0.538
2	*EEF1B2*	67	206732562	206735898	0.747	0.444	0.829
2	*INPP4A*	117	98427844	98570598	0.138	0.89	0.338
2	*PECR*	130	216611355	216654777	0.221	0.379	0.146
3	*CRBN*	132	3166695	3196390	0.883	0.299	0.508
4	*CCNA2*	74	122957048	122964538	**0.016**	0.054	**0.016**
4	*LOC90826*	55	148778982	148824730	0.482	0.116	0.221
4	*PRSS12*	134	119421864	119493370	0.06	0.404	0.253
5	*NDST1*	99	149880622	149917966	0.661	0.185	0.753
6	*ASCC3*	404	101063328	101435945	0.128	0.575	0.456
6	*GRIK2*	868	101953625	102624651	0.435	0.775	0.802
6	*MED23*	104	131936798	131991056	0.982	0.598	0.94
7	*CASP2*	69	142695523	142714907	0.885	0.502	0.96
8	*C8orf41*	109	33475777	33490245	**0.014**	0.313	**0.041**
8	*NIBP*	738	140811769	141537860	0.732	0.851	0.685
8	*TUSC3*	490	15442100	15666366	0.105	0.441	0.129
9	*C9orf86*	57	138822201	138855460	0.979	0.822	0.982
9	*MAN1B1*	41	139101199	139123460	0.947	0.809	0.925
9	*RALGDS*	104	134962927	135014409	0.893	0.995	0.992
10	*ADK*	376	75580970	76139066	0.983	0.312	0.996
10	*ENTPD1*	220	97461525	97627013	0.642	0.455	0.444
11	*C11orf46*	110	30301224	30315741	0.680	0.663	0.375
12	*ASCL1*	11	101875581	101878424	0.950	0.05	0.508
12	*COQ5*	66	119425464	119451347	0.553	0.397	0.445
12	*CRADD*	239	92595281	92768662	0.485	0.239	0.272
12	*KIAA1033*	159	104025621	104087036	0.567	0.265	0.427
12	*ZCCHC8*	41	121523387	121551471	0.549	0.204	0.326
14	*UBR7*	91	92743153	92765314	0.751	0.886	0.703
14	*ZC3H14*	71	88099066	88149606	0.904	0.292	0.585
15	*SCAPER*	298	74427591	74963247	0.298	0.051	**0.037**
16	*PRRT2*	17	29730909	29734703	0.393	0.471	0.705
17	*FASN*	52	77629502	77649395	0.514	0.276	0.510
18	*ELP2*	144	31963884	32008605	**0.044**	0.123	0.248
19	*CC2D1A*	35	13878051	13902692	0.657	0.722	0.734
19	*GPSN2*	68	14501381	14537792	0.074	0.223	0.104
19	*TRMT1*	32	13076714	13088332	0.633	0.811	0.657
19	*ZNF526*	16	47416331	47424193	0.509	0.595	0.627

Three genes were nominally associated with fluid ability, one was nominally associated with crystallised ability, and four with general cognitive ability. Start and end positions do not include the ± 50 kb boundary. Bold indicates nominally significant (p < 0.05) genes.

**Table 2:** This shows the statistically significant keywords describing the shared biological functions of the NS-ARID genes indicating the systems they are found in. This set was ascertained through an automatic literature search implemented in GRAIL.

Keywords	Gene symbols
Synthase (2 genes)	*ST3GAL3*, *ELP2*
Reductase (3 genes)	*FASN*, *ENTPD1*, *ADK*
Mitochondrial (7 genes)	*CASP2*, *ECR*, *CRADD*, *RABL6*, *TECR*, *ADK*, *FASN*
Apoptosis (7 genes)	*RABL6*, *ELP2*, *FASN*, *ENTPD1*, *HIST3H3*, *MED23*, *ADK*
Methyltransferase (1 gene)	*HIST3H3*
Elegans (11 genes)	*CASP2*, *TRMT1*, *TUSC3*, *SCAPER*, *RABL6*, *ASCC3*, *TRAPPC9*, *ADK*, *PRSS12*, *CRADD*, *EEF1B2*
Complex (12 genes)	*MED23*, *EEF1B2*, *CRADD*, *HIST3H3*, *CASP2*, *SCAPER*, *TRAPPC9*, *TECR*, *ASCC3*, *MAN1B1*, *RALGDS*, *TTI2*
Death (4 genes)	*TRAPPC9*, *FASN*, *ENTPD1*, *NDST1*
Genome (10 genes)	*TRAPPC9*, *ELP2*, *TECR*, *RABL6*, *TRMT1*, *COQ5*, *EEF1B2*, *PRSS12*, *TUSC3*, *MED23*
Histone (2 genes)	*CC2D1A*, *MED23*
Enzyme (12 genes)	*ADK*, *TUSC3*, *ST3GAL3*, *PECR*, *PRSS12*, *MAN1B1*, *FASN*, *ENTPD1*, *CASP2*, *TECR*, *SCAPER*, *HIST3H3*
Trna (3 genes)	*ELP2*, *TUSC3*, *TECR*
Adenosine (1 gene)	*NDST1*
Elongation (5 genes)	*ELP2*, *PECR*, *MED23*, *RALGDS*, *TUSC3*
Fatty (3 genes)	*PECR*, *ADK*, *CASP2*
Saccharomyces (9 genes)	*ELP2*, *ASCC3*, *MAN1B1*, *COQ5*, *EEF1B2*, *TECR*, *ADK*, *NDST1*, *MED23*
Cerevisiae (9 genes)	*TRMT1*, *ASCC3*, *MAN1B1*, *COQ5*, *EEF1B2*, *TECR*, *ADK*, *NDST1*, *MED23*
Yeast (13 genes)	*COQ5*, *EEF1B2*, *TRMT1*, *ASCC3*, *RABL6*, *TECR*, *MAN1B1*, *MED23*, *RALGDS*, *SCAPER*, *HIST3H3*, *TRAPPC9*, *NDST1*

Abbreviation: NS-ARID, non-syndromic autosomal recessive intellectual disability. Trna, Transfer Ribonucleic acid.

**Table 3:** The five most significant Gene Ontology gene sets for the functional gene group analysis and their association with fluid cognitive ability.

GO term	Name	Number of genes		p-value		Genes in LD independent intervals
Total	N hit	Enrichment	Corrected
GO:0006814	Sodium ion transport	165	11	7.9e^− 5^	0.014	*SLC10A7*, *SLC8A1*, *SLC5A1*, *SLC4A5*, *SLC4A10*, *ACCN1*, *SLC9A10*, *SLC9A9*, *SLC17A8*, *NEDD4L*, *SLC34A2*
GO:0055029	Nuclear DNA-directed RNA polymerase complex	97	4	0.015	0.685	*SUPT3H*, *POLR2E*, *POLR2B*, *POLR3F*
GO:0030880	RNA polymerase complex	98	4	0.015	0.695	*SUPT3H*, *POLR2E*, *POLR2B*, *POLR3F*
GO:0006354	DNA-templated transcription, elongation	85	3	0.023	0.810	*POLR2E*, *POLR2B*, *POLR3F*
GO:0016591	DNA-directed RNA polymerase II, holoenzyme	86	3	0.059	0.958	*POLR2E*, *POLR2B*, *POLR3F*

Number of genes total pertains to the full number of genes in the gene set. Number of genes N hit indicates how many of the independent intervals overlapped with the genes of the gene set. Abbreviations: GO, Gene Ontology.

**Table 4:** The five most significant Gene Ontology gene sets for the functional gene group analysis and their association with crystallised cognitive ability.

GO term	Name	Number of genes		p-value		Genes in LD independent intervals
Total	N hit	Enrichment	Corrected
GO:0032781	Positive regulation of ATPase activity	19	3	0.002	0.174	*TPM1*, *RYR2*, *UHRF1*
GO:0006353	DNA-templated transcription, termination	82	4	0.003	0.218	*DHX38*, *CCNH*, *POLR3B*, *POLR2E*
GO:0006354	DNA-templated transcription, elongation	85	4	0.004	0.299	*CCNH*, *POLR3B*, *POLR2E*, *POLR2B*
GO:0043462	Regulation of ATPase activity	29	3	0.004	0.302	*TPM1*, *RYR2*, *UHRF1*
GO:0006368	Transcription elongation from RNA polymerase II promoter	65	3	0.012	0.569	*CCNH*, *POLR2E*, *POLR2B*

Number of genes total pertains to the full number of genes in the gene set. Number of genes N hit indicates how many of the independent intervals overlapped with the genes of the gene set. Abbreviations: GO, Gene Ontology.

**Table 5:** The five most significant Gene Ontology gene sets for the functional gene group analysis and their association with general cognitive ability.

GO term	Name	Number of genes		p-value		Genes in LD independent intervals
Total	N hit	Enrichment	Corrected
GO:0005665	Positive regulation of ATPase activity	15	2	0.005	0.481	*POLR2E*, *POLR2B*
GO:0043462	DNA-templated transcription, termination	28	3	0.005	0.481	*TPM1*, *PLN*, *UHRF1*
GO:0035098	DNA-templated transcription, elongation	12	2	0.005	0.537	*EED*, *MTF2*
GO:0031062	Regulation of ATPase activity	17	2	0.012	0.795	*EED*, *MTF2*
GO:0031060	Transcription elongation from RNA polymerase II promoter	24	2	0.028	0.942	*EED*, *MTF2*

Number of genes total pertains to the full number of genes in the gene set. Number of genes N hit indicates how many of the independent intervals overlapped with the genes of the gene set. Abbreviations: GO, Gene Ontology.
