# Supplementary material for: Examining non-syndromic autosomal recessive intellectual disability (NS-ARID) genes for an enriched association with intelligence differences
Source: Intelligence. 2016 Jan-Feb;54:80–9. doi: 10.1016/j.intell.2015.11.005 (PMC4725222; doi:10.1016/j.intell.2015.11.005)
Supplement: Supplementary material — Results using the gene set first analysed by Franić, et al. (2015). S1 shows the results of SNP based analysis, S2 and Table S1 show the results of gene-based analysis. S3 shows the results of the Franić, et al. (2015) gene-set when considering all the genes as the unit of association. [file mmc1.docx]

**Supplementary results**

**S1. Single Marker analysis**

None of the 4,211 SNPs tested in either the fluid ability or crystallised cognitive phenotypes attained statistical significance where α= 1.187366e-05. Supplementary Figure 1 shows no deviation from that which would be expected under the null hypothesis.


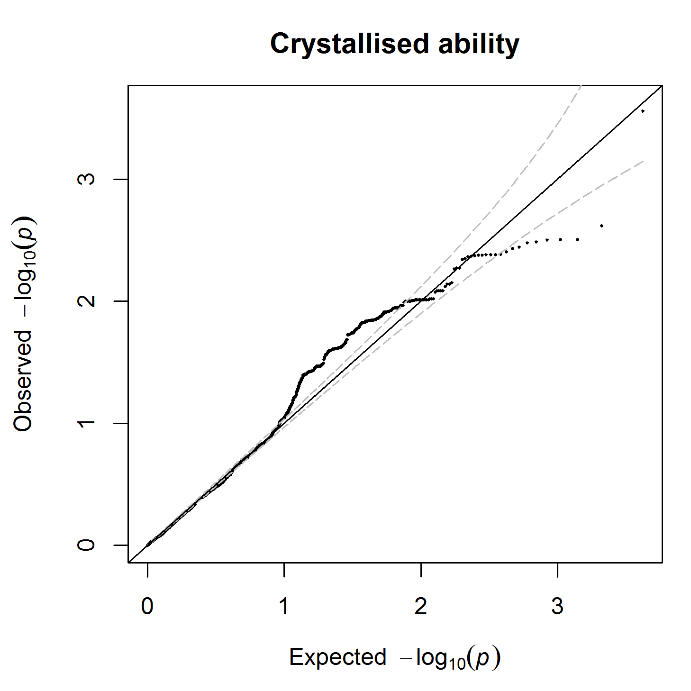

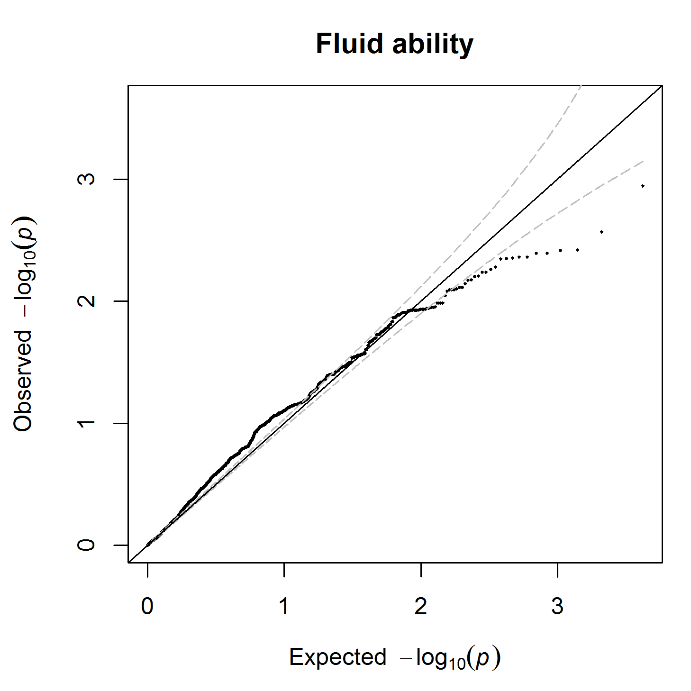


Figure S1. These qq plots show the full complement of 4,211 SNPs in from the gene-set of [Franić, et al. (2015)](#_ENREF_16) both fluid ability (left) and crystallised ability (right). These plots indicate that for both phenotypes there is no deviation from that which would be expected under the null hypothesis of no association.

**S2. Gene-based analysis**

VEGAS ([Liu, et al., 2010](#_ENREF_32)) was used to examine the contribution each gene in the gene set used by ([Franić, et al., 2015](#_ENREF_16)) made to both fluid and crystallised ability. No single gene-based statistic was significant at the Bonferroni adjusted alpha level of 0.001. Four nominally significant genes were found for *gf* (*SLC31A1, C8orf41, CCNA2, ELP2*) and Two for crystallised ability (*POLR3B, SLC31A1*); these results are consistent with what would be expected under the null hypothesis.

Table S1. Gene based analysis results for the [Franić, et al. (2015)](#_ENREF_16) genes in the CAGES consortium.

| Chr | Gene | nSNPs | Start | Stop | Fluid P-value | Crystallised P-value |
| --- | --- | --- | --- | --- | --- | --- |
| 1 | *ACBD6* | 271 | 178523987 | 178738102 | 0.230 | 0.731 |
| 1 | *HIST3H3* | 48 | 226679168 | 226679649 | 0.203 | 0.734 |
| 1 | *ZBTB40* | 105 | 22650930 | 22730237 | 0.669 | 0.389 |
| 1 | *CNKSR1* | 107 | 26376567 | 26388962 | 0.690 | 0.174 |
| 1 | *PARP1* | 134 | 224615014 | 224662424 | 0.717 | 0.286 |
| 1 | *GON4L* | 46 | 153986133 | 154093596 | 0.823 | 0.799 |
| 1 | *RGS7* | 804 | 239005439 | 239587101 | 0.983 | 0.997 |
| 2 | *INPP4A* | 117 | 98427844 | 98570598 | 0.138 | 0.89 |
| 2 | *CAPN10* | 118 | 241174817 | 241205795 | 0.184 | 0.827 |
| 2 | *PECR* | 130 | 216611355 | 216654777 | 0.221 | 0.379 |
| 2 | *EEF1B2* | 67 | 206732562 | 206735898 | 0.747 | 0.444 |
| 4 | *CCNA2* | 74 | 122957048 | 122964538 | **0.016** | 0.054 |
| 4 | *LOC90826* | 55 | 148778982 | 148824730 | 0.482 | 0.116 |
| 5 | *NDST1* | 99 | 149880622 | 149917966 | 0.661 | 0.185 |
| 6 | *ASCC3* | 404 | 101063328 | 101435945 | 0.128 | 0.575 |
| 6 | *HIST1H4B* | 65 | 26135102 | 26135459 | 0.648 | 0.425 |
| 7 | *CASP2* | 69 | 142695523 | 142714907 | 0.885 | 0.502 |
| 8 | *C8orf41* | 109 | 33475777 | 33490245 | **0.014** | 0.313 |
| 8 | *TAF2* | 208 | 120812194 | 120914255 | 0.133 | 0.378 |
| 8 | *ERLIN2* | 14 | 37713254 | 37734477 | 0.325 | 0.100 |
| 9 | *SLC31A1* | 172 | 115023688 | 115066593 | **0.006** | **0.047** |
| 9 | *RALGDS* | 104 | 134962927 | 135014409 | 0.893 | 0.995 |
| 9 | *MAN1B1* | 41 | 139101199 | 139123460 | 0.947 | 0.809 |
| 9 | *C9orf86* | 57 | 138822201 | 138855460 | 0.979 | 0.822 |
| 10 | *ENTPD1* | 220 | 97461525 | 97627013 | 0.642 | 0.455 |
| 10 | *ADK* | 376 | 75580970 | 76139066 | 0.983 | 0.312 |
| 11 | *TMEM135* | 510 | 86426712 | 86712218 | 0.114 | 0.435 |
| 11 | *C11orf46* | 110 | 30301224 | 30315741 | 0.680 | 0.663 |
| 12 | *POLR3B* | 213 | 105275618 | 105428105 | 0.107 | **0.008** |
| 12 | *ZCCHC8* | 41 | 121523387 | 121551471 | 0.549 | 0.204 |
| 12 | *JARID1A* | 162 | 259483 | 368881 | 0.814 | 0.920 |
| 12 | *ASCL1* | 11 | 101875581 | 101878424 | 0.950 | 0.05 |
| 13 | *FRY* | 426 | 31503436 | 31768776 | 0.421 | 0.837 |
| 14 | *UBR7* | 91 | 92743153 | 92765314 | 0.751 | 0.886 |
| 15 | *SCAPER* | 298 | 74427591 | 74963247 | 0.298 | 0.051 |
| 15 | *KIF7* | 86 | 87972210 | 87992578 | 0.592 | 0.320 |
| 15 | *LINS1* | 118 | 98926957 | 98959927 | 0.759 | 0.761 |
| 17 | *CACNA1G* | 125 | 45993447 | 46059541 | 0.338 | 0.734 |
| 17 | *FASN* | 52 | 77629502 | 77649395 | 0.514 | 0.276 |
| 17 | *WDR45L* | 105 | 78165726 | 78199700 | 0.791 | 0.873 |
| 17 | *JMJD3* | 59 | 7683959 | 7698843 | 0.865 | 0.811 |
| 18 | *ELP2* | 144 | 31963884 | 32008605 | **0.044** | 0.123 |
| 18 | *LAMA1* | 375 | 6931885 | 7107813 | 0.235 | 0.285 |

Four genes were nominally associated with fluid ability and two were nominally associated with crystallised ability. Start and end positions do not include the ±50 kb boundary. Bold indicates nominally significant (p <0.05) genes.

**S3. Gene-set analysis**

INRICH was performed on the same gene set used by [Franić, et al. (2015)](#_ENREF_16) using the same 5kb boundary. The results with the fluid cognitive phenotype in the CAGES sample found overlap with only one gene *C8orf41* (p=1) where for crystallised cognitive ability there was overlap with one gene *POLR3B* this overlap was also not significant, p=0.533.

These results follow those in the main body of the text that the genes involved in intellectual disabilities are not enriched for variants that are associated with the normal range of intelligence differences.
